# Health insurance and social capital in Ghana: a cluster randomised controlled trial

**DOI:** 10.1186/s41256-018-0090-y

**Published:** 2018-12-06

**Authors:** Christine J. Fenenga, Katalin Buzasi, Daniel K. Arhinful, Stephen K. O. Duku, Alice Ogink, Wouter Poortinga

**Affiliations:** 1 Amsterdam Institute for Global Health and Development, Paasheuvelweg 24, 1105 BP Amsterdam, The Netherlands; 2 University of Groningen, Broerstraat 5, 9712 CP Groningen, The Netherlands; 3 Noguchi Memorial Institute for Medical Research, College of Health Sciences, University of Ghana, Legon, Accra, Ghana; 4 Vrije Universiteit Amsterdam, De Boelelaan 1105, 1081 HV Amsterdam, The Netherlands; 5 Tinbergen Institute, Gustav Mahlerplein 117, 1082 MS Amsterdam, The Netherlands; 6 Cardiff University, Cardiff, CF10 3AT Wales, UK

**Keywords:** Ghana, Social capital, Health insurance, Marginal effects, Cluster randomised controlled trial, Systematic client engagement

## Abstract

**Background:**

The National Health Insurance Scheme (NHIS) was introduced in Ghana in 2003, enrolment is still far from the desired target of universal coverage. Low community engagement in the design and management of the system was identified as one of the main barriers. The aim of the current study was to explore the role of social capital in NHIS enrolment in two regions of Ghana, Western and Greater Accra.

**Methods:**

The study involved a cluster-randomised controlled trial of 3246 clients of 64 healthcare facilities who completed both a baseline and a follow-up survey. Thirty-two facilities were randomly selected to receive two types of intervention. The remaining facilities served as control. The interventions were co-designed with stakeholders. Baseline and follow up surveys included measures of different types of social capital, as well as enrolment in the health insurance scheme.

**Results:**

The study found that the interventions encouraged NHIS enrolment (from 40.29 to 49.39% (intervention group) versus 36.49 to 36.75% (control group)). Secondly, certain types of social capital are associated with increased enrolment (log-odds ratios (*p*-values) of three types of vertical social capital are 0.127 (< 0.01), 0.0952 (< 0.1) and 0.15 (< 0.01)). Effectiveness of the interventions was found dependent on initial levels of social capital: respondents with lowest measured level of interpersonal trust in the intervention group were about 25% more likely to be insured than similar respondents in the control group. Among highly trusting respondents this difference was insignificant. There was however no evidence that the interventions effect social capital. Limitations of the study are discussed.

**Conclusion:**

We showed that the interventions helped to increase enrolment but that the positive effect was not realized by changes in social capital that we hypothesised based on result of the first phase of our study. Future research should aim to identify other community factors that are part of the enrolment process, whether other interventions to improve the quality of services could help to increase enrolment and, as a result, could provide community benefits in terms of social capital.

Our findings can guide the NHIS in Ghana and other health organizations to enhance enrolment.

**Trial registration:**

Ethical Clearance by Ghana Health Service Ethical Committee No. GHS-ERC 08.5.11.

**Electronic supplementary material:**

The online version of this article (10.1186/s41256-018-0090-y) contains supplementary material, which is available to authorized users.

## Introduction

Ghana introduced the National Health Insurance Scheme (NHIS) in 2003 to improve access to universal quality healthcare. It replaced a cash and carry system that required upfront payment from individuals at the point of service usage. Membership of the NHIS provide access to health services from both credentialed public and private healthcare providers throughout the country, and are financially covered for about 95% of Ghana’s health problems. Members pay an annual premium for active membership unless they fall under one of several exemption categories [1]. The scheme increased the number of people with access to health insurance, but enrolment levels have stayed well below the desired universal coverage target. A decade after its implementation, only about 34% of Ghana’s population were active card bearing members [2–4]. Several barriers to enrolment have been identified, relating to the scheme’s affordability, the supply of NHIS membership cards, poor attitudes of healthcare staff, perceived preferential treatment for fee-paying patients, and substandard information provision [5–10]. In addition, communities with low levels of social trust and trust in healthcare service have been found to have below average active membership [8], suggesting that social capital may play role in NHIS enrolment in Ghana.

Social capital is a multifaceted concept that has been used extensively in health research over the past two decades [11–14]. Social capital can be defined in different ways [15, 16], but is in the health literature mostly used as an indication of ‘*social cohesion*’ – whereby social capital is seen as a property of groups, with resources such as trust, norms and the exercise of sanctions, available to individual members in that group; or of a ‘*network’* – whereby resources, such as social support, information channels, social credentials, are embedded within an individual’s social network [17]. Szreter and Woolcock [18] distinguish between different types of social capital, i.e. *bonding*, *bridging* and *linking,* reflecting different social links that exist in society. While *Bonding* social capital reflect ties between individuals with a relatively high degree of network closure, such as in families, close relatives, friends, neighbors, often associated with strong norms and trust, *Bridging* social capital concerns ties between individuals across social and economic divides, or between groups or associations, such as trade unions, professional groups, women groups. Bridging social capital may not involve many shared norms but is often associated with reciprocity and ‘thin trust’ [16]. In contrast to bonding and bridging social capital, which are both seen as reflecting horizontal social ties within and across social groups, *linking* social capital (sometimes referred to as *vertical social capital*) concerns norms of respect and networks of trusting relationships across explicit, formal, or institutionalized power or authority gradients in society [16, 18].

Like in many African countries, social capital, in particular bonding social capital, is believed to be an important aspect of community life in Ghana [19, 20]. Strong bonding ties exist in the extended families, where patriarchs lead important decisions [19]. Further, well-organized community groups, such as women groups, church communities, professional groups, and saving groups, exist in both urban and rural areas. These bonding community networks of trust and reciprocity play an important role in individual day-to-day decision-making, including health decisions [21, 22], and have been linked to a series of health outcomes [23–26, 27, 28]. The question is whether social capital may also be linked to other health seeking behaviors, including enrolment in health insurance schemes.

This paper reports on the study on enrollment in the NHIS in Ghana, and the role of social capital therein. The study consisted of a *cluster randomized controlled trial* to test two social-capital informed interventions to increase enrolment (see section “Methods” below). The interventions were innovative in that it aimed to promote health insurance enrollment through increasing social capital.

**Fig. 1:**
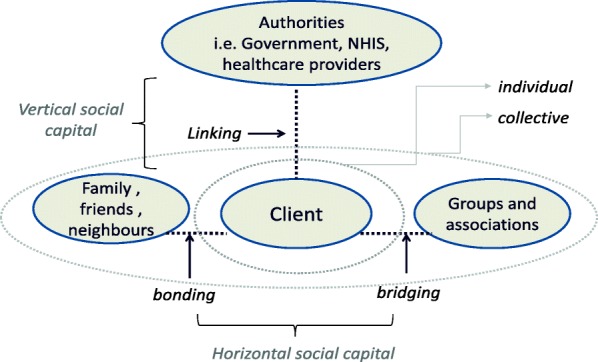
Conceptual social capital framework (Fenenga et al. [8])

The study has shown in a previous publication that there are high levels of social capital in Ghana, and identified different types of social capital [8]. In particular, it found two types horizontal social capital, reflecting trust and solidarity, and collective action within the community; and two types of vertical social capita, reflecting trust in the healthcare provider and the NHIS respectively (Fig. 1).

Community trust and solidarity, as well as trust in the healthcare provider and health insure were all associated with active enrollment in the NHIS. Based on these results, two social-capital informed interventions were developed with the intention to increase enrolment (see section “Design of interventions”).

In the current paper we report the results of the randomized controlled trial. In particular, we examined (1) the effectiveness of the interventions on active membership in the NHIS, (2) whether the interventions helped to increase levels of social capital, and (3) whether the effectiveness of the interventions was dependent on initial levels of social capital. We expected that 1) the interventions increase enrollment in the NHIS; 2) the interventions increase levels of social capital; and 3) the effectiveness of the intervention depends on initial levels of social capital. In addition, we expect that 4) the interventions will be the most effective n communities with initial low levels of trust and solidarity and where community members are less likely to encourage each other to enroll,

## Methods

This clustered randomized controlled trial was conducted in 2011–2014 among NHIS insured and non-insured clients of primary healthcare facilities in the predominantly urban region Greater Accra and the more rural Western Region in Ghana.

### Sampling

The study used a multi-stage sampling strategy. The first stage of the multi-stage sampling strategy was the purposive selection of 16 NHIS district schemes (8 in each region) based on total population, NHIS enrolment coverage, NHIA accreditation status, and geographical location (urban – rural). In the second stage, 64 primary healthcare facilities (32 in each region) were selected on the basis of their ownership (public/private), location (rural/urban), and NHIS accreditation quality scores. A third stage of sampling was conducted to collect data from 1903 randomly selected households in both regions. Thirty households were sampled from within a radius of 10 km around each of the 64 selected primary healthcare facilities. In total data of 7097 individuals in the sampled households was collected in the baseline survey, which took place in April 2012.

For the intervention, 32 health facilities (from the 64) were randomly selected to receive the interventions, while the remaining 32 facilities served as controls without an intervention [29–31]. A follow-up survey was conducted among 6971 individuals from the 64 facilities, which took place between March and June 2014 after the interventions were completed.

### Design of interventions

The two interventions were designed through an iterative participatory process based on the results of the baseline survey. Stakeholders (the healthcare clients, healthcare providers, and the National Health Insurance Authority (NHIA)) gave their input to derive to the key components for the intervention [8]. The participatory approach, whereby stakeholders co-design the intervention, was chosen to facilitate interaction, mutual learning, empowerment and trust-building between the different stakeholder groups. The rationale of co-design is that stakeholders know their context and interest best and can help define an appropriate, acceptable and feasible intervention which has a better chance to achieve result. Both interventions were also based on the concept of social capital.

**Fig. 2:**
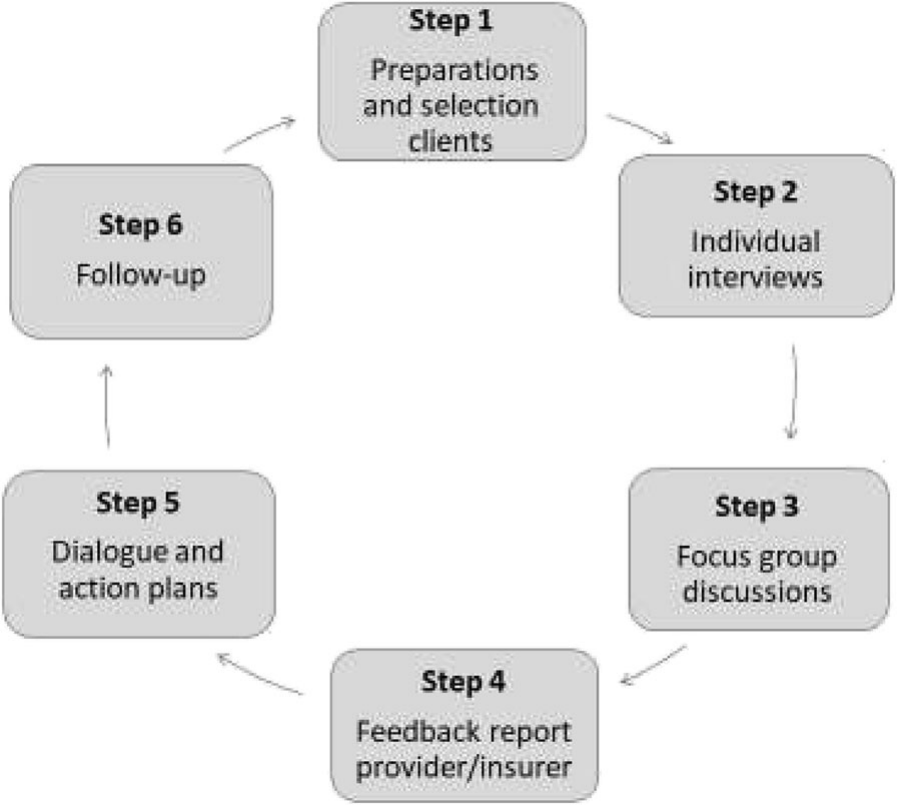
The six steps of the intervention design

*Intervention 1* (the light version), conducted in 26 facilities, engaged *existing community groups*, identified with help of the district authorities, to monitor healthcare and health insurance services and suggest improvements (See step by step process below and Fig. 2). This process was facilitated by a trained community health worker (CHW). The intervention included also two meetings between the community groups and the healthcare provider and district NHIS staff to discuss monitoring results and suggestions for improvement. We reason this process *builds social capital* (*horizontal)* by giving an important role to the group in improving health services in their community. Through information sharing and discussions between the group members, the CHW and the healthcare provider and NHIS staff, *social capital (vertical)* can be built.

*Intervention 2* (the intensive version), conducted in 6 facilities, engaged *individual clients*, identified by the CHW during their visit to the clinic. The CHW subsequently visited the individual client at his/her home *within 6 days, assuring a short recall-period* that would lead to more accurate responses than in intervention 1 where group members of existing groups may not have used services for some time. Recall periods in health have been studied by other researchers [32, 33]. To minimize recall bias one should consider methodological approaches such as using well-structured standardized questions [32]. For intervention 2 we used structured questions and locally produced illustrative pictures. We adopted the conceptual model of the validated assessment test used in TB-care [34]. This TB Quote instrument uses a 6 days recall period. This period is also practical: it allows the community worker to follow the patient in the community more easily than when using a period of a month or longer. Secondly, it allows the project to proceed, analyse and validate the data and design an improvement plan with the stakeholders.

The second interventions also included group meetings of the same clients that were first interviewed individually. In the group they discussed their monitoring findings and suggestions with healthcare and district NHIS staff. We reasoned that although this intervention may tap slightly less on the horizontal social capital (engaging individuals and not existing community groups), it does stimulate empowerment and strengthens vertical social capital through the interaction with the health facility and NHIS staff.

Both interventions thus entailed participatory monitoring of services and service improvement. In the monitoring, participants focused on ten service aspects (indicators) that derived from the baseline study in which we explored clients’ definition and perception on quality of services. Related to the healthcare provider these aspects were 1) Attitude of staff; 2) Punctuality of staff; 3) Information provision; 4) Availability of drugs; 5) Queuing system; 6) Opportunity to provide feedback. For the NHIS these were: 7) Information provision; 8) Enrollment process; 9) Delivering services that are promised; 10) Opportunity to provide feedback. We applied a mixed methodology of scoring, using a scale from 1 (poor) to 5 (excellent), and narrative responses. In addition, the Net Promotor Score (NPS) was used to measure the willingness of clients to promote the services (of health care provider or NHIS) to neighbors and friends, using a scale of 1 (very unlikely) to 4 (very likely).

Intervention 2 comprised of six steps as presented in Fig. 2, while the intervention 1 comprised of five steps, making intervention 2 more intensive than intervention 1*.* Both interventions were implemented and evaluated concurrently in the period May 2013 to February 2014. Results of these interventions are published by Alhassan et al. [29], Duku et al. [30] and Fenenga et al. [31] and. The following section briefly describes the engagement steps.

*Step 1* involved the recruitment of healthcare clients at the exit of a healthcare facility; *Step 2* involved individual face-to-face interviews with clients in their own homes to obtain information about their experiences and views on healthcare facilities and health insurance. The results from the interviews were used as basis for a focus group discussion in *Step 3*. The focus group discussions were used to share information among group members about the assessment of the 10 mentioned aspects and NPS, but also to validate the findings of Step 2. Focus group discussions are usually more dynamic and generate new information through action-reaction responses. *Step 4* consisted of written and verbal reports to provide feedback to healthcare providers and district insurance offices. A joint stakeholder meeting was convened in *Step 5*. The meetings served as an open dialogue forum, involving different stakeholders to develop action plans to improve the services and make them more client focused. The final step (*Step 6*) was included to evaluate the implemented actions. Plaques of honor and small financial incentives were used to reward improved services of the healthcare provider and district NHIS office.

### Respondents and measures

As discussed above, the number of respondents were 7097 and 6971 in the baseline and follow-up surveys, respectively. In total, 3246 individuals were identified who filled out both surveys and were above the age of 18. Additional file 1: Figure A.1 presents a flow diagram showing the inclusion of observations in the final sample. As Additional file 1: Table A.1 suggests, respondents removed from the sample are largely similar to those included in the final sample. There are slight differences in terms of education profile and experience with paying for consultation and drugs. The only major difference is that the majority of removed individuals were from the Greater Accra region.

While there were two types of interventions, they were combined for the analyses because they produced similar effects (the analyses with the two interventions separately, with essentially similar results to the analysis with the combined group are presented in Additional file 1). Table 1 shows that the control and the combined intervention group of the final sample differ in a number of aspects. The Western region is somewhat underrepresented in the control group. The share of respondents evaluating their health status worse compared to other people similar to them is higher in the control group, while the probability of considering health status the same is higher in the intervention group. People in the control group were more likely to have paid for consultation in the past. These factors were included in the statistical models as controls.

**Table 1:** Intervention group characteristics at baseline

	Control group	Intervention group (combined)	*P*-value
Age (in years)	37.74	37.81	0.896
Sex (% women)	55.80	57.05	0.474
Region (% Western region)	46.78	52.11	0.002***
Religion (% Christian)	90.00	89.88	0.155
Household wealth			
Wealth quintile 1 (%)	16.65	17.71	0.902
Wealth quintile 2 (%)	17.40	17.41	
Wealth quintile 3 (%)	19.55	18.73	
Wealth quintile 4 (%)	22.89	20.84	
Wealth quintile 5 (%)	23.52	25.3	
Highest level of completed education			
No formal education (%)	12.98	13.77	0.139
Less than primary school (%)	3.74	2.55	
Primary school (%)	9.94	9.28	
Middle/junior secondary school (%)	39.14	40.45	
Secondary/senior secondary school (%)	21.72	19.35	
Vocational/polytechnical training (%)	7.54	8.79	
Higher education (%)	4.94	5.82	
Is your health better or worse than of other people of the same sex and age in the community			
Better (%)	61.62	59.6	0.01**
The same (%)	34.35	37.48	
Worse (%)	4.02	2.92	
The share of respondents paying for consultation and drugs during last healthcare provider visit			
Pay for consultation (%)	21.56	18.67	0.04**
Pay for drugs (%)	47.23	46.87	0.838

Note: The *p*-values refer to the results of the chi-square test, except for age where independent t-test with unequal assumed variances is used; * *p* < 0.05; ** *p* < 0.01; *** *p* < 0.001

The main *outcome variable* of this study was enrolment in any health insurance scheme (survey question: “Are you currently enrolled in any health insurance scheme?”). A descriptive analysis on this variable is found in Section “The impacts of the intervention on social capital”.

*Social capital* was measured by items consisting of 16 statements. Respondents were asked to what extent they agreed or disagreed with the statements. They could respond using a five-point Likert scale from strongly disagree (with the value of 1) to strongly agree (with the value of 5). The items were developed using the theoretical framework as outlined in Fenenga et al. [8], covering the topics of trust, solidarity, community action, and trust in healthcare and NHIS services. A principal components analysis with Varimax rotation found that five social capital factors accounted for 66.8% of the original variance in the data (Additional file 1: Table B.1). The first factor comprised items related to trust in people in the community. This factor was labelled “*horizontal social capital – trust*” (HC_trust), reflecting bonding social capital (see Fig. 1). The second factor, labelled “*vertical social capital – provider 1*” (VC_prov1) contained items related to attitudes to personnel and fairness of the queuing system in the facility. The third factor was labelled “*Horizontal social capital – action*” (HC_action), and can be considered to reflect aspects of both bonding and bridging capital (see Fig. 1). The fourth factor, labelled “*Vertical social capital – provider 2*” (VC_prov2) reflects views on the quality of the facilities. The last factor “Ver*tical social capital – NHIS*” (VC_nhis) captures the trustworthiness and adequacy of the National Health Insurance Scheme. The items and factor loading are presented in Additional file 1: Table B.2.

The *socio-demographic sections of the questionnaire* included questions on age, sex, region, religion, household wealth, and highest level of completed education. Household wealth is measured as the annual per capita food and non-food consumption expenditure. The questionnaire further included question on health status (“*Is your health better or worse than that of other people of the same sex and age you know in your community?*”), and experiences with healthcare provider at the last visit (“*The last time you visited a health provider, did you have to pay (out of pocket) for consultation, tests or lab services?*” and “*Did you pay for drugs when you visited this facility?*”). The questionnaire and items are described in more detail in Additional file 1: Table A.2.

### Model specifications

We used multilevel modelling to analyse the data consisting of individuals clustered within facilities. First, we constructed a set of two multilevel logit models to examine the effectiveness of the intervention in terms of enrolment in the NHIS insurance scheme. The first model included a dummy indicating the measurement occasion (follow-up versus baseline) and a dummy indicating the intervention group. The relative changes in the intervention group as compared to the control group were indicated by the interaction term of the two. The second model further included the five social capital factors as described above.

Second, we constructed a series of five multilevel regression models in which we used the social capital factors as the dependent variables. Just as for the first set of analyses, we included dummies indicating the measurement occasion, the intervention group, and the interaction between measurement occasion and the intervention group. Controls were also included.

Third, we constructed a series of five multilevel models to determine whether the effectiveness of the interventions was dependent on initial levels of social capital. The dependent variable was enrolment in insurance at follow-up. The models contain insurance enrolment at baseline, a dummy for the intervention group and the socio-demographic controls. The variables of interest were the interactions between the intervention group and social capital aspects. Instead of presenting full tables, we report only the marginal effects of the intervention group (compared to control) at different initial social capital levels.

## Results

### The effectiveness of the intervention

**Fig. 3:**
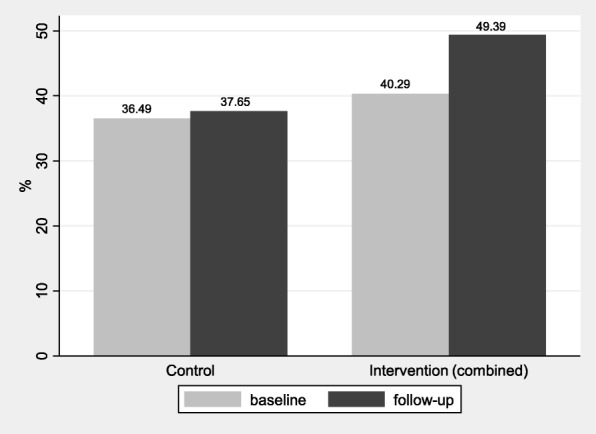
Health insurance in the control and the combined intervention group

Figure 3 shows the differences of insurance coverage in the control and the combined intervention group at baseline and follow-up. The share of respondent with insurance coverage grew from 36.49 to 36.75% in the control group and from 40.29 to 49.39% in the (combined) intervention group. According to the independent t-test, the 4 percentage point higher insurance coverage of the intervention group at baseline is significant at the 5% level (ΔM = − 3.80, *p* = 0.029). The difference between the control and intervention group increased to about 12 percentage point by the time of the follow-up survey and the difference is significant at the 1% level (ΔM = − 11.74, *p* = 0.000). Changes over time were assessed with paired t-tests. While the small increase in the control group is statistically not significant (ΔM = 1.16, *p* = 0.424), the about 9 percentage point change in the treatment group is significant at the 1% level (ΔM = 9.10, *p*-value = 0.000). Figure 3 suggests that the intervention promoted insurance enrollment.

The conclusion of Fig. 3 is confirmed by the first set of multilevel regressions in Table 2, with a significant interactions between the intervention and follow-up (Model 1). The interaction term remained significant when controlling for the different social capital variables (Model 2 in Table 2). The significant coefficients of VC_prov1 VC_prov2 and VC_nhis show that vertical social capital plays an important role in enrolment across the two waves of data collection. The full model with controls (Additional file 1: Table C.1).

**Table 2:** The effect of the intervention on insurance enrolment and the relationship between social capital and insurance

Dependent variable:	Model 1	Model 2
	Being ensured	Being ensured
Intervention (IG)	0.176 (0.142)	0.143 (0.135)
Follow-up	−0.254** (0.124)	−0.153 (0.129)
IG x Follow-up	0.414** (0.164)	0.454*** (0.160)
HC_trust		0.00775 (0.0466)
HC_action		0.0460 (0.0404)
VC_prov1		0.127*** (0.0442)
VC_prov2		0.0952* (0.0489)
VC_nhis		0.150*** (0.0510)
Constant	−2.928*** (0.259)	−3.004*** (0.263)
Variance of intercept	0.18*** (0.043)	0.165*** (0.042)
Observations	6007	5895
Number of groups	64	64
Log pseudolikelihood	− 3556.8111	− 3461.7883

Note: errors are clustered by facility; controls listed in Table 1 are included *HC_trust 1* Horizontal social capital – trust, *HC_action* Horizontal social capital – action, *VC_prov1* Vertical social capital – provider 1, *VC_prov2* Vertical social capital – provider 2, *VC_nhis* Vertical social capital – NHIS; * *p* < 0.05; ** *p* < 0.01; *** *p* < 0.001

### The impacts of the intervention on social capital

**Fig. 4:**
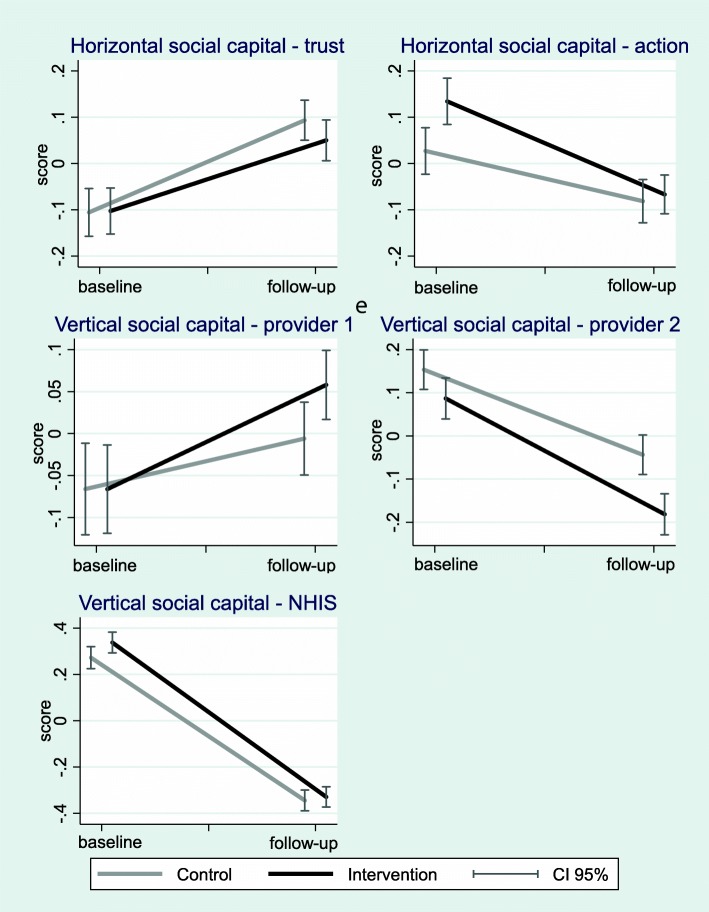
Changes in the five social capital factors from baseline to follow-up. Notes: The levels (scores) of social capital are estimated from 16 survey items with principal component analysis explained in Section “Respondents and measures” and Additional file 1

Figure 4 illustrates the level (score) of the five types of social capital estimated with principal component analysis (explained in Section “Respondents and measures”. and Additional file 1) at baseline and follow up for the control and intervention group.

While the “Horizontal social capital – trust” and “Vertical social capital - provider 1” increased for the two intervention groups, “Horizontal social capital – action”, “Vertical social capital - provider 2” and “Vertical social capital - NHIS” decreased from baseline to follow up. In addition, the control groups appear to show similar patterns as the two intervention groups which indicates that changes in social capital between the two survey years are not influenced by the intervention.

Table 3 shows the results of the multilevel regression analyses, reflecting the main patterns found in Fig. 3 there was an overall increase in “Horizontal social capital – trust” (HC trust), and a general decrease in “Vertical social capital – provider 2” (VC prov2) and “Vertical social capital - NHIS” (VC nhis). The intervention group dummy is included to capture any significant differences compared to Control at baseline. We find no significant differences in social capital between the control and intervention group at baseline.

**Table 3:** The effect of the interventions on the level of social capital aspects

Dependent variable:	Model 1	Model 2	Model 3	Model 4	Model 5
	HC_trust	HC_action	VC_prov1	VC_prov2	VC_nhis
Follow-up	0.227*** (0.0736)	− 0.0997 (0.0812)	0.0712 (0.0753)	− 0.150** (0.0702)	− 0.634*** (0.0796)
Intervention group (IG)	−0.0224 (0.0820)	0.111 (0.0803)	−0.0106 (0.0861)	− 0.0279 (0.110)	0.0616 (0.0719)
IG x Follow-up	−0.0847 (0.108)	− 0.124 (0.108)	0.0515 (0.0999)	− 0.0642 (0.114)	−0.0610 (0.100)
Constant	−0.269* (0.146)	0.0544 (0.116)	−0.301** (0.141)	0.264** (0.132)	0.251*** (0.0967)
Variance of intercept	0.057*** (0.015)	0.068*** (0.014)	0.038*** (0.008)	0.193*** (0.038)	0.042*** (0.009)
Observations	6013	6013	6013	6013	6013
Number of groups	64	64	64	64	64
Log pseudolikelihood	− 8107.2474	− 8346.0905	− 8445.4805	− 7753.767	− 8101.5881

Note: errors are clustered by facility, controls listed in Table 1 are included; *** *p* < 0.01, ** *p* < 0.05, * *p* < 0.1

The interaction terms between the follow-up and intervention group dummy were non-significant in all models, suggesting that the intervention had no differential effect on any of the social capital factors. In summary, the similar social capital change patterns across the control and intervention group are confirmed by the multivariate regression models. The full table with the coefficients of socio-demographic controls is presented in Additional file 1: Table C.2.

### Initial levels of social capital and the effectiveness of the intervention

**Fig. 5:**
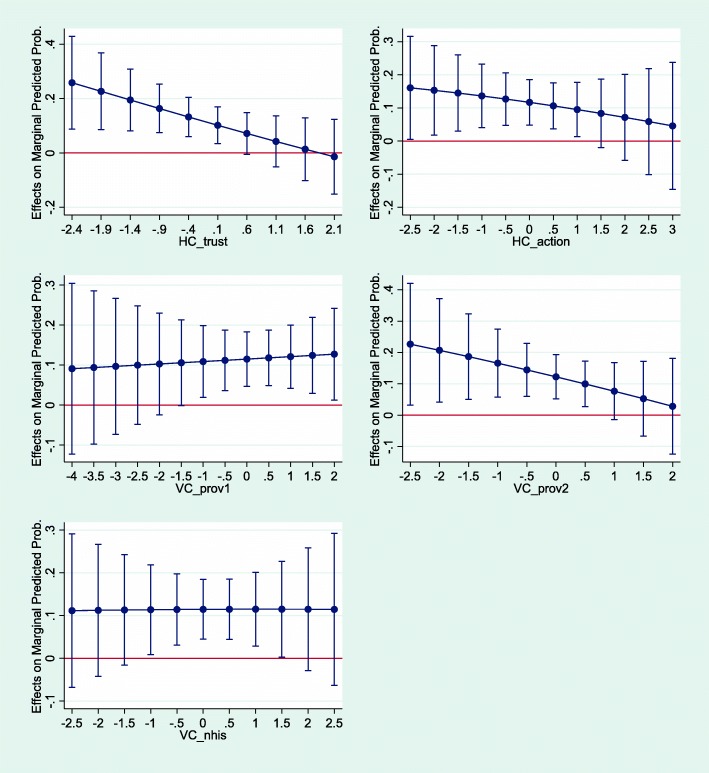
The marginal effects of the interventions on enrolment by initial social capital level. Note: Marginal effects are based on models in Additional file 1: Table C.3 containing interaction terms

We subsequently conducted a series of analyses introducing interaction terms between the initial levels of social capital types and the intervention group types to explore whether the effectiveness of the interventions is dependent on social capital. Each model controls for being insured at baseline, the intervention group and a set of socio-demographic controls. Moreover, each model contains the baseline level of one of the social capital aspects and their interaction with the intervention group. All remaining variables are kept at their mean. The full regression table is reported in Additional file 1: Table C.3; here (Fig. 5) we present only the marginal effects estimated from Additional file 1: Table C.3. The lines in Fig. 5 can be interpreted as follows. Each circle of the line indicates how much higher or lower the probability of a respondent being insured in the intervention group compared to a similar other in the control group at various initial social capital levels.

The general pattern suggests that the effectiveness of the intervention is greater at lower levels of “horizontal social capital – trust”, “horizontal social capital – action” and “vertical social capital – provider 2”. It also shows that beyond a certain level, the effects are independent of a person receiving the intervention or not. An individual with high levels of social capital in the intervention group then would have the same chance of enrolling in insurance as in the control group. “Vertical social capital – provider 1” seems to influence the effectiveness of the intervention the opposite way. If we moved two people from control to the intervention group, the likelihood of enrolling into insurance of the one with higher “Vertical social capital – provider 1” would be increased to a greater extent.

## Discussion

The sustainability of health insurance schemes that currently arise in many low and middle-income countries to improve access to quality health care and lower the out-of-pocket expenses is challenged by a range of factors. These have been well described in the literature [35–39]. One important factor is realizing adequate enrolment and retention of clients. In order to explore solutions that will support Ghana’s NHIS to increase its sustainability, this study explored whether and how client engagement would be an effective strategy.

While various studies have demonstrated the potential value of social capital in influencing health seeking behaviour [23, 25], this study builds upon this knowledge by testing whether client engagement in monitoring and improving healthcare and health insurance services positively influence clients’ trust in the NHIS and their willingness to enrol in the program. Reasoning that stakeholder participation in the design and implementation of the interventions will strengthen communication, information sharing, mutual understanding and relationship building, we hypothesized that social capital (horizontal and vertical) and subsequently insurance enrolment would increase as a result of the intervention.

This paper presents the results of a *cluster randomised controlled trial* in which two interventions were tested to increase active membership of the Ghana NHIS. The study examined whether (1) the interventions were effective at raising active membership in the NHIS, (2) the interventions helped to increase levels of social capital, and (3) the effectiveness of the interventions was dependent on initial levels of social capital. As the effects of the two interventions were comparable, they were combined in the analyses. Other papers published on these interventions focus on the effect on patient safety and risk reduction efforts in primary health facilities [29] and on healthcare utilization, frequency of illness and perceptions on quality of healthcare [30]. Our first main finding is that the combined intervention was effective in increasing NHIS enrolment. The second main finding is the positive relationship between the vertical types of social capital and insurance enrolment. It reinforces previous findings in the literature [40, 41] that trustworthiness of providers and insurance schemes and positive attitudes of medical staff play an important role in people’s decision on joining insurance schemes. The third main finding is that the interventions are more effective where there is a low level of social capital. In practice, it means that in a low social capital environment even light engagement interventions might be a cost-effective tool in increasing insurance enrolment.

While we have shown that the interventions effectively increased enrolment, and that social capital has a positive effect on enrolment, findings are inconclusive about the effect of the interventions on the different types of social capital. In other words, we can show that the combined intervention had a positive effect on enrolment but not that it affects social capital. Future research should aim to identify other community factors that are part of the enrolment process, whether other interventions to improve the quality of services could help to increase enrolment and, as a result, could provide community benefits in terms of social capital. Our baseline and follow-up survey include a range of social capital items, which could potentially be linked to the concept of social capital. Another direction is to identify and test potential channels other than social capital through which the intervention could impact enrolment. One explanation of the effectiveness of the intervention can be the increased attention on health insurance, encouraging healthcare and health insurance providers to improve their services. Alhassan et al. concluded from this same study that healthcare staff efforts towards increasing patient safety and reducing risk improved significantly in intervention facilities especially in the areas of leadership/accountability [29].

## Conclusion

In conclusion we can say that the intervention increases enrolment in the health insurance program. Although we did not find a change in social capital, we reason that the intervention effect of improved services in time will also influence clients’ trust in the service providers and thus increase the level of vertical social capital. It would be reasonable that such change require longer continuation of the intervention.

Finally, findings can guide the NHIS in Ghana and other countries to realize more client engagement in the planning and implementation of their programs in order to enhance enrolment.

## Additional file

Additional file 1: Respondent inclusion. (DOCX 111 kb)

## Abbreviations

HC: Horizontal social capital
NDPC: National Demographic Planning Commission
NHIA: National Health Insurance Authority
NHIS: National Health Insurance Scheme
VC: Vertical social capital
