# Supplementary material for: Health insurance and social capital in Ghana: a cluster randomised controlled trial
Source: Glob Health Res Policy. 2018 Dec 6;3:35. doi: 10.1186/s41256-018-0090-y (PMC6282266; doi:10.1186/s41256-018-0090-y)
Supplement: Supplementary file 1 — Respondent inclusion. (DOCX 111 kb) [file 41256_2018_90_MOESM1_ESM.docx]

**Additional file 1**

**Respondent inclusion**

**Figure A.1** Inclusion in final sample

*Follow up*

6,971

Control: 3,448

Combined intervention: 3,523

*Baseline*

7,097

Control: 3,588

Combined intervention: 3,509

*Matched individuals*

5,451

Control: 2,680

Combined intervention: 2,771

*Matched individuals aged 18 or above at baseline*

3,246

Control: 1,586

Combined intervention: 1,660

**Table A.1** Comparison of the final sample with removed observations (at baseline)

|  | Removed observations  (above 18 years old) | Remaining observations | Difference |
| --- | --- | --- | --- |
| **Insured** (%) | 42.56 | 40.97 | 0.015 |
| **Age** (in years) | 37.63 | 37.77 | -0.141 |
| **Sex** (% women) | 55.79 | 56.44 | -0.6 |
| **Region** (% Western region) | 29.68 | 49.51 | -19.83*** |
| **Religion** (% Christian) | 89.05 | 89.77 | -0.7 |
| **Household wealth** |  |  |  |
| Wealth quintile 1 (%) | 22.23 | 19.87 | 2.36 |
| Wealth quintile 2 (%) | 15.24 | 20.46 | -5.21*** |
| Wealth quintile 3 (%) | 23.59 | 18.42 | 5.17*** |
| Wealth quintile 4 (%) | 16.7 | 20.33 | -3.63*** |
| Wealth quintile 5 (%) | 22.23 | 20.92 | 1.31 |
| **Highest level of completed education** |  |  |  |
| No formal education (%) | 13.03 | 13.38 | -0.35 |
| Less than primary school (%) | 3.93 | 3.13 | 0.8 |
| Primary school (%) | 9.62 | 9.6 | 0.02 |
| Middle/junior secondary school (%) | 37.95 | 39.81 | -1.86 |
| Secondary/senior secondary school (%) | 19.65 | 20.51 | -0.86 |
| Vocational/polytechnical training (%) | 9.81 | 8.13 | 1.68 |
| Higher education (%) | 5.99 | 5.36 | 0.6 |
| **Is your health better or worse than of other people of the same sex and age in the community** |  |  |  |
| Better (%) | 60.34 | 60.88 | -0.54 |
| The same (%) | 34.39 | 33.88 | 0.5 |
| Worse (%) | 5.27 | 5.24 | 0.03 |
| **The share of respondents paying for consultation and drugs during last healthcare provider visit** |  |  |  |
| Pay for consultation (%) | 16.92 | 20.09 | -3.17*** |
| Pay for drugs (%) | 21.12 | 29.11 | -7.99**** |

**Table A.2.** Description of the socio-demographic variables

| **Survey item** | **Original answer categories** | **Description** | **Name in tables (empirical analysis)** |
| --- | --- | --- | --- |
| B4: What was your/ [NAME]’s age at last birthday? | - | Continuous | age |
| B2: Sex | 1. Male, (1) Female | Categorical | female (reference category: man) |
| wealth quintile | 1. wealth quintile 1, (2) wealth quintile 2, (3) wealth quintile 3, (4) wealth quintile 4, (5) wealth quintile 5 | Categorical. Individuals are assigned into five wealth groups of equal size based on the annual per capita food and non-food household consumption. | wealth quintile 2, 3, 4 and 5 (reference category: wealth quintile 1) |
| B7: What is your / [NAME]’s highest level of completed education? | 1. None, (2) Less than primary, (3) Primary, (4) Middle/JSS, (5) Secondary/SSS, (6) Tech/Vocational, (7) Polytechnic, (8) First degree, (9) Postgraduate | Categorical. Due to small number of responses, category 6 is combined with category 7, and category 8 is combined with category 9. | some primary educ, primary educ, junior secondary, senior secondary, vocational or polytechnical (combined 6&7), higher educ (combined 8&9) (reference category: no education) |
| B6: What is your/[NAME]’s religion? | 1. Christian, (2) Muslim, (3) Traditional, (4) None, (5) Other | Categorical. Due to small number of respondents, categories (2) to (5) are combined. | Christian (reference category: all other, combined 2&3&4&5) |
| region | 1. Greater Accra, (2) Western | Categorical. | Western region (reference category: Greater Accra) |
| E3: Is your health better or worse than that of other people of the same sex and age that you know in your community? | 1. Much better, (2) Better, (3) Same, (4) Worse, (5) Much worse | Categorical. (1) and (2) are combined as Better, (4) and (5) combined as Worse. | health status=same, health status=worse (reference category: better) |
| E12: The last time you/[NAME] visited a health provider did you/he/she have to pay (out of pocket cash) for consultation, tests or laboratory services at this facility? | 1. Yes, (2) No | Categorical. | Pay for consultation (reference category: No) |
| E22: Did you/ [NAME] pay for drug that you/he/she received at the facility during this visit? | 1. Yes, (2) No | Categorical. | Pay for drugs (reference category: No) |

**Constructing social capital with factor analysis**

To construct the social capital factors (types) we used the following survey questions

*How much do you agree with the following statements? Possible answers: (1) strongly disagree, (2) disagree, (3) neither, (4) agree, (5) strongly agree*

i1a) I trust most people in this community (SC1)

i1c) I trust my village elders (SC2)

i1d) I trust my assembly man/woman (SC3)

i2a) Most people in this community help others when they are in need (SC4)

i2c) People in this community will contribute money to projects even if they have no direct benefit from it (SC5)

i2f) People in this community will collaborate to solve any health service related problem (SC6)

g2a) The doctors, medical assistants and nurses are compassionate and very supportive (SC7)

g2b) The doctors, medical assistants and nurses treated me respectfully (SC8)

g2b) There are sufficiently good doctors, medical assistants and nurses (SC9)

g2d2) I received most prescribed drugs from the facility (SC10)

g2e1) There is a fair and well organized queuing system in the facility (SC11)

g2e2) There are adequate consulting room and medical equipment (SC12)

g2f) Health personnel treats patients with insurance cards in an equal way as patients without insurance card (SC13)

h1a) The information from media on NHIS is adequate (SC14)

h1b1) The NHIS is trustworthy (SC15)

h1b2) The services covered by NHIS are adequate (SC16)

**Table B.1** Factor analysis (rotated)

**Table B.2** Factor loadings

| Survey item | Factor 1 | Factor 2 | Factor 3 | Factor 4 | Factor 5 |
| --- | --- | --- | --- | --- | --- |
| I trust most people in this community | ***0.83*** | 0.02 | 0.25 | 0.01 | 0.04 |
| I trust my village elders | ***0.9*** | 0.05 | 0.16 | 0.01 | 0.03 |
| I trust my Assembly man/woman | ***0.83*** | 0.07 | 0.16 | 0.02 | 0.1 |
| Most people in this community help others when they are in need | 0.38 | 0.03 | ***0.72*** | 0.04 | 0.02 |
| People in this community will contribute money to projects even if they have no direct benefit from it | 0.21 | 0.01 | ***0.84*** | 0.05 | 0.09 |
| People in this community will collaborate to solve any health service related problem | 0.17 | 0.06 | ***0.83*** | 0.04 | 0.11 |
| The doctors, medical assistants and nurses are compassionate and very supportive | 0.05 | ***0.87*** | 0.05 | 0.15 | 0.02 |
| The doctors, medical assistants and nurses treated me respectfully | 0.04 | ***0.88*** | 0.03 | 0.17 | 0.07 |
| There are sufficiently good doctors, medical assistants and nurses | -0.01 | 0.26 | 0.08 | ***0.78*** | 0.06 |
| I received most prescribed drugs from the facility | 0.08 | 0.18 | 0.05 | ***0.68*** | 0.12 |
| There is a fair and well organized queuing system in the facility | 0.09 | ***0.63*** | 0.0 | 0.25 | 0.04 |
| There are adequate consulting room and medical equipment | 0.0 | 0.15 | 0.03 | ***0.85*** | 0.15 |
| Health personnel treats patients with insurance cards in an equal way as patients without insurance card | 0.01 | 0.33 | 0.21 | 0.22 | 0.35 |
| The information from media on NHIS is adequate | -0.03 | 0.01 | 0.12 | 0.05 | ***0.74*** |
| The NHIS is trustworthy | 0.15 | 0.06 | 0.05 | 0.11 | ***0.77*** |
| The services covered by NHIS are adequate | 0.09 | 0.08 | 0.1 | 0.18 | ***0.78*** |

Identified social capital types

Factor 1 – Horizontal social capital – trust

Factor 3 – Horizontal social capital – action

Factor 2 – Vertical social capital – provider 1

Factor 4 – Vertical social capital – provider 2

Factor 5 – Vertical social capital – NHIS

Analysis with the combined intervention group

**Table C.1** Intervention effect on insurance (full table)

|  | Model 1 | Model 2 |
| --- | --- | --- |
|  | Being insured | Being insured |
|  |  |  |
| follow-up | -0.254** | -0.153 |
|  | (0.124) | (0.129) |
| intervention group (IG) | 0.176 | 0.143 |
|  | (0.142) | (0.135) |
| follow-up#IG | 0.414** | 0.454*** |
|  | (0.164) | (0.160) |
| HC_trust |  | 0.00775 |
|  |  | (0.0466) |
| HC_action |  | 0.0460 |
|  |  | (0.0404) |
| VC_prov1 |  | 0.127*** |
|  |  | (0.0442) |
| VC_prov2 |  | 0.0952* |
|  |  | (0.0489) |
| VC_nhis |  | 0.150*** |
|  |  | (0.0510) |
| female | 0.563*** | 0.562*** |
|  | (0.0701) | (0.0710) |
| age | 0.0217*** | 0.0213*** |
|  | (0.00229) | (0.00228) |
| wealth quintile 2 | 0.317*** | 0.349*** |
|  | (0.115) | (0.117) |
| wealth quintile 3 | 0.284** | 0.338** |
|  | (0.131) | (0.132) |
| wealth quintile 4 | 0.416*** | 0.466*** |
|  | (0.153) | (0.158) |
| wealth quintile 5 | 0.663*** | 0.737*** |
|  | (0.139) | (0.147) |
| Western region | 0.599*** | 0.567*** |
|  | (0.124) | (0.125) |
| less than primary | -0.0857 | -0.0743 |
|  | (0.188) | (0.197) |
| primary | -0.153 | -0.152 |
|  | (0.129) | (0.129) |
| junior secondary | 0.176 | 0.164 |
|  | (0.115) | (0.118) |
| senior secondary | 0.576*** | 0.568*** |
|  | (0.110) | (0.116) |
| vocational/polytechnical | 0.653*** | 0.652*** |
|  | (0.147) | (0.151) |
| higher education | 0.541*** | 0.542** |
|  | (0.207) | (0.212) |
| pay for consultation | -1.167*** | -1.135*** |
|  | (0.111) | (0.111) |
| Christian | 0.113 | 0.120 |
|  | (0.157) | (0.156) |
| health status=same | 0.0310 | 0.0452 |
|  | (0.0878) | (0.0888) |
| health status=worse | 0.549*** | 0.556*** |
|  | (0.148) | (0.146) |
| pay for drugs | 0.697*** | 0.692*** |
|  | (0.0874) | (0.0870) |
| Constant | -2.928*** | -3.004*** |
|  | (0.259) | (0.263) |
|  |  |  |
| Variance of intercepts | 0.18*** | 0.165*** |
|  | (0.043) | (0.042) |
| Observations | 6,007 | 5,895 |
| Number of groups | 64 | 64 |
| Log pseudo likelihood | -3556.8111 | -3461.7883 |

Note: This table is the full version of Table 2. The dependent variable is being insured. Errors are clustered by facility. * p<0.05; ** p<0.01; *** p<0.001.

**Table C.2** Intervention effect on social capital (full table)

|  | (1) | (2) | (3) | (4) | (5) |
| --- | --- | --- | --- | --- | --- |
|  | HC_trust | HC_action | VC_prov1 | VC_prov2 | VC_NHIS |
|  |  |  |  |  |  |
| follow-up | 0.227*** | -0.0997 | 0.0712 | -0.150** | -0.634*** |
|  | (0.0736) | (0.0812) | (0.0753) | (0.0702) | (0.0796) |
| intervention group (IG) | -0.0224 | 0.111 | -0.0106 | -0.0279 | 0.0616 |
|  | (0.0820) | (0.0803) | (0.0861) | (0.110) | (0.0719) |
| follow-up#IG | -0.0847 | -0.124 | 0.0515 | -0.0642 | -0.0610 |
|  | (0.108) | (0.108) | (0.0999) | (0.114) | (0.100) |
| female | -0.0744*** | 0.00213 | -0.00757 | 0.0263 | -0.00948 |
|  | (0.0232) | (0.0245) | (0.0208) | (0.0202) | (0.0222) |
| age | 0.00149* | -0.000230 | 0.00229** | -0.000101 | 0.000924 |
|  | (0.000768) | (0.000926) | (0.000934) | (0.000819) | (0.000805) |
| wealth quintile 2 | 0.0320 | 0.0700 | -0.0493 | 0.0278 | 0.0282 |
|  | (0.0887) | (0.0608) | (0.0667) | (0.0596) | (0.0641) |
| wealth quintile 3 | -0.0175 | 0.0851 | 0.0169 | -0.00471 | -0.0142 |
|  | (0.0930) | (0.0684) | (0.0859) | (0.0707) | (0.0693) |
| wealth quintile 4 | 0.0175 | 0.0112 | -0.0880 | -0.0973 | 0.0191 |
|  | (0.0906) | (0.0618) | (0.0800) | (0.0652) | (0.0705) |
| wealth quintile 5 | -0.108 | 0.0793 | -0.151* | -0.0808 | -0.0118 |
|  | (0.0923) | (0.0671) | (0.0850) | (0.0766) | (0.0752) |
| Western region | 0.475*** | 0.150** | 0.133** | -0.213* | 0.117** |
|  | (0.0659) | (0.0712) | (0.0558) | (0.113) | (0.0561) |
| less than primary | 0.113 | 0.104 | -0.0324 | -0.123* | 0.111* |
|  | (0.0764) | (0.0987) | (0.0816) | (0.0645) | (0.0657) |
| primary | -0.123** | -0.0223 | 0.0216 | -0.0301 | 0.0310 |
|  | (0.0586) | (0.0629) | (0.0542) | (0.0526) | (0.0525) |
| junior secondary | -0.0700 | -0.0290 | -0.00954 | -0.0249 | 0.0134 |
|  | (0.0559) | (0.0478) | (0.0437) | (0.0416) | (0.0410) |
| senior secondary | -0.136** | -0.0613 | -0.00646 | -0.0266 | 0.00536 |
|  | (0.0657) | (0.0597) | (0.0668) | (0.0520) | (0.0567) |
| vocational/polytechnical | -0.152** | 0.00602 | -0.0513 | -0.0560 | 0.00848 |
|  | (0.0679) | (0.0779) | (0.0682) | (0.0685) | (0.0630) |
| higher education | -0.0763 | -0.0467 | -0.119 | -0.128 | -0.332*** |
|  | (0.107) | (0.0955) | (0.113) | (0.0810) | (0.0920) |
| Christian | -0.100 | -0.0495 | 0.107 | -0.0474 | -0.0377 |
|  | (0.0672) | (0.0754) | (0.0863) | (0.0620) | (0.0539) |
| pay for consultation | 0.0465 | -0.0265 | -0.0715* | 0.00356 | -0.191*** |
|  | (0.0448) | (0.0428) | (0.0411) | (0.0385) | (0.0415) |
| health status=same | 0.113** | -0.164*** | 0.0494 | 0.00621 | 0.0413 |
|  | (0.0446) | (0.0443) | (0.0423) | (0.0420) | (0.0466) |
| health status=worse | 0.0509 | -0.0533 | 0.0500 | -0.0480 | 0.137* |
|  | (0.0724) | (0.0742) | (0.0715) | (0.0716) | (0.0791) |
| pay for drugs | 0.153*** | -0.0462 | 0.135*** | 0.219*** | -0.00161 |
|  | (0.0394) | (0.0433) | (0.0340) | (0.0423) | (0.0345) |
| Constant | -0.269* | 0.0544 | -0.301** | 0.264** | 0.251*** |
|  | (0.146) | (0.116) | (0.141) | (0.132) | (0.0967) |
|  |  |  |  |  |  |
| Variance of intercepts | 0.057*** | 0.068*** | 0.038*** | 0.193*** | 0.042*** |
|  | (0.015) | (0.014) | (0.008) | (0.038) | (0.009) |
| Observations | 6,013 | 6,013 | 6,013 | 6,013 | 6,013 |
| Number of groups | 64 | 64 | 64 | 64 | 64 |
| Log pseudolikelihood | -8107.2474 | -8346.0905 | -8445.4805 | -7753.767 | -8101.5881 |

Note: This table is the full version of Table 3. Dependent variables are shown in column heading by model specification. Errors are clustered by facility. HC_trust 1 = Horizontal social capital – trust, HC_action = Horizontal social capital – action, VC_prov1 = Vertical social capital – provider 1, VC_prov2 = Vertical social capital – provider 2, VC_nhis = Vertical social capital – NHIS; * p<0.05; ** p<0.01; *** p<0.001

**Table C.3** Interaction effects: intervention and the initial levels of social capital (full table)

|  | (1) | (2) | (3) | (4) | (5) |
| --- | --- | --- | --- | --- | --- |
|  | insured at follow-up | insured at follow-up | insured at follow-up | insured at follow-up | insured at follow-up |
|  |  |  |  |  |  |
| insured at baseline | 1.480*** | 1.489*** | 1.491*** | 1.493*** | 1.490*** |
|  | (0.180) | (0.181) | (0.180) | (0.181) | (0.180) |
| Intervention group (IG) | 0.535*** | 0.579*** | 0.569*** | 0.611*** | 0.574*** |
|  | (0.173) | (0.177) | (0.175) | (0.181) | (0.182) |
| HC_trust | 0.0638 | -0.0885 | -0.0862 | -0.0851 | -0.0859 |
|  | (0.103) | (0.0838) | (0.0837) | (0.0831) | (0.0838) |
| IG#HC_trust | -0.290* |  |  |  |  |
|  | (0.161) |  |  |  |  |
| HC_action | 0.107 | 0.177 | 0.114 | 0.119 | 0.115 |
|  | (0.0760) | (0.119) | (0.0754) | (0.0758) | (0.0758) |
| IG#HC_action |  | -0.124 |  |  |  |
|  |  | (0.150) |  |  |  |
| VC_prov1 | 0.0321 | 0.0345 | 0.0188 | 0.0298 | 0.0316 |
|  | (0.0649) | (0.0651) | (0.102) | (0.0643) | (0.0659) |
| IG#VC_prov1 |  |  | 0.0249 |  |  |
|  |  |  | (0.130) |  |  |
| VC_prov2 | 0.0563 | 0.0559 | 0.0528 | 0.181 | 0.0523 |
|  | (0.0944) | (0.0936) | (0.0936) | (0.133) | (0.0941) |
| IG#VC_prov2 |  |  |  | -0.238 |  |
|  |  |  |  | (0.183) |  |
| VC_nhis | 0.107 | 0.105 | 0.103 | 0.102 | 0.110 |
|  | (0.0852) | (0.0851) | (0.0859) | (0.0843) | (0.137) |
| IG#VC_nhis |  |  |  |  | -0.0164 |
|  |  |  |  |  | (0.176) |
| Western region | 0.434** | 0.444*** | 0.437*** | 0.429** | 0.436*** |
|  | (0.169) | (0.167) | (0.168) | (0.172) | (0.169) |
| age | 0.0182*** | 0.0177*** | 0.0178*** | 0.0177*** | 0.0178*** |
|  | (0.00381) | (0.00381) | (0.00379) | (0.00385) | (0.00385) |
| female | 0.430*** | 0.424*** | 0.423*** | 0.423*** | 0.423*** |
|  | (0.103) | (0.104) | (0.105) | (0.104) | (0.104) |
| wealth quintile 2 | -0.0920 | -0.113 | -0.102 | -0.108 | -0.0982 |
|  | (0.176) | (0.176) | (0.175) | (0.177) | (0.173) |
| wealth quintile 3 | -0.126 | -0.122 | -0.118 | -0.107 | -0.116 |
|  | (0.220) | (0.216) | (0.216) | (0.215) | (0.216) |
| wealth quintile 4 | -0.0382 | -0.0446 | -0.0355 | -0.0342 | -0.0340 |
|  | (0.230) | (0.227) | (0.230) | (0.228) | (0.229) |
| wealth quintile 5 | -0.134 | -0.140 | -0.130 | -0.138 | -0.129 |
|  | (0.238) | (0.238) | (0.242) | (0.240) | (0.239) |
| pay for consultation | -1.361*** | -1.372*** | -1.375*** | -1.386*** | -1.375*** |
|  | (0.211) | (0.213) | (0.212) | (0.209) | (0.214) |
| pay for drugs | 0.979*** | 1.005*** | 0.999*** | 1.003*** | 1.000*** |
|  | (0.176) | (0.176) | (0.176) | (0.176) | (0.176) |
| health status=same | 0.111 | 0.108 | 0.109 | 0.114 | 0.111 |
|  | (0.140) | (0.137) | (0.137) | (0.135) | (0.137) |
| health status=worse | 0.561* | 0.546* | 0.550* | 0.561* | 0.553* |
|  | (0.295) | (0.294) | (0.294) | (0.292) | (0.292) |
| less than primary | -0.229 | -0.243 | -0.232 | -0.233 | -0.233 |
|  | (0.264) | (0.264) | (0.265) | (0.266) | (0.264) |
| primary | -0.0878 | -0.0925 | -0.0897 | -0.0818 | -0.0888 |
|  | (0.237) | (0.237) | (0.236) | (0.235) | (0.236) |
| junior secondary | 0.166 | 0.165 | 0.167 | 0.166 | 0.167 |
|  | (0.171) | (0.171) | (0.170) | (0.170) | (0.170) |
| senior secondary | 0.358* | 0.354* | 0.356* | 0.349* | 0.356* |
|  | (0.192) | (0.191) | (0.191) | (0.189) | (0.192) |
| vocational/polytechnical | 0.750*** | 0.761*** | 0.754*** | 0.746*** | 0.755*** |
|  | (0.236) | (0.233) | (0.234) | (0.232) | (0.234) |
| higher education | -0.168 | -0.123 | -0.137 | -0.136 | -0.136 |
|  | (0.393) | (0.393) | (0.392) | (0.393) | (0.391) |
| Christian | -0.119 | -0.128 | -0.133 | -0.136 | -0.136 |
|  | (0.257) | (0.257) | (0.260) | (0.255) | (0.258) |
| Constant | -3.025*** | -3.021*** | -3.017*** | -3.036*** | -3.018*** |
|  | (0.457) | (0.456) | (0.457) | (0.453) | (0.462) |
|  |  |  |  |  |  |
| Variance of intercepts | 0.319*** | 0.329 | 0.336*** | 0.347*** | 0.336*** |
|  | (0.068) | (0.073) | (0.072) | (0.078) | (0.073) |
| Observations | 2,723 | 2,723 | 2,723 | 2,723 | 2,723 |
| Number of groups | 64 | 64 | 64 | 64 | 64 |
| Log pseudolikelihood | -1405.6195 | -1408.9411 | -1409.7017 | -1407.4747 | -1409.7256 |

Note: The dependent variable of each model specification is being insured at follow-up. This table is the basis for Figure 5. Dependent variables are shown in column heading by model specification. Errors are clustered by facility. HC_trust 1 = Horizontal social capital – trust, HC_action = Horizontal social capital – action, VC_prov1 = Vertical social capital – provider 1, VC_prov2 = Vertical social capital – provider 2, VC_nhis = Vertical social capital – NHIS; * p<0.05; ** p<0.01; *** p<0.001

**Additional tables**

**Table D.1** The effect of the intervention on insurance enrolment and the relationship between social capital and insurance (intervention groups are treated separately)

|  | (1) | (2) |
| --- | --- | --- |
|  | Being insured | Being insured |
|  |  |  |
| follow up (post-treatment) | -0.254** | -0.154 |
|  | (0.124) | (0.129) |
| Intervention group 1 | 0.0863 | 0.0550 |
|  | (0.145) | (0.139) |
| Intervention group 2 | 0.558** | 0.519*** |
|  | (0.222) | (0.193) |
| follow up#IG1 | 0.444** | 0.489*** |
|  | (0.179) | (0.172) |
| follow up#IG2 | 0.281* | 0.302** |
|  | (0.156) | (0.133) |
| HC_trust |  | 0.0103 |
|  |  | (0.0464) |
| HC_action |  | 0.0471 |
|  |  | (0.0403) |
| VC_prov1 |  | 0.128*** |
|  |  | (0.0443) |
| VC_prov2 |  | 0.0951* |
|  |  | (0.0487) |
| VC_nhis |  | 0.148*** |
|  |  | (0.0509) |
| female | 0.561*** | 0.560*** |
|  | (0.0702) | (0.0711) |
| age | 0.0218*** | 0.0213*** |
|  | (0.00230) | (0.00228) |
| wealth quintile 2 | 0.315*** | 0.347*** |
|  | (0.115) | (0.117) |
| wealth quintile 3 | 0.278** | 0.332** |
|  | (0.131) | (0.132) |
| wealth quintile 4 | 0.413*** | 0.463*** |
|  | (0.153) | (0.158) |
| wealth quintile 5 | 0.657*** | 0.732*** |
|  | (0.139) | (0.147) |
| Western region | 0.597*** | 0.563*** |
|  | (0.122) | (0.122) |
| Christian | 0.110 | 0.117 |
|  | (0.158) | (0.156) |
| some primary educ | -0.0835 | -0.0724 |
|  | (0.188) | (0.197) |
| primary educ | -0.154 | -0.154 |
|  | (0.129) | (0.129) |
| junior secondary | 0.176 | 0.164 |
|  | (0.115) | (0.118) |
| senior secondary | 0.572*** | 0.564*** |
|  | (0.110) | (0.116) |
| vocational or polytechnical | 0.652*** | 0.650*** |
|  | (0.146) | (0.151) |
| higher educ | 0.548*** | 0.548*** |
|  | (0.206) | (0.211) |
| paid for consultation | -1.167*** | -1.136*** |
|  | (0.111) | (0.112) |
| health status=same | 0.0297 | 0.0443 |
|  | (0.0877) | (0.0886) |
| health status=worse | 0.549*** | 0.556*** |
|  | (0.147) | (0.145) |
| paid for drugs | 0.696*** | 0.690*** |
|  | (0.0875) | (0.0869) |
| Constant | -2.917*** | -2.992*** |
|  | (0.260) | (0.264) |
|  |  |  |
| Observations | 6,007 | 5,895 |
| Number of groups | 64 | 64 |

Note: The dependent variable is being insured (shown in column headings). Errors are clustered by facility. * p<0.05; ** p<0.01; *** p<0.001.

**Table D.2**. Intervention effect on social capital (intervention groups are treated separately)

|  | Model 1 | Model 2 | Model 3 | Model 4 | Model 5 |
| --- | --- | --- | --- | --- | --- |
|  | HC_trust | HC_action | VC_prov1 | VC_prov2 | VC_nhis |
|  |  |  |  |  |  |
| follow-up | 0.227*** | -0.0998 | 0.0712 | -0.150** | -0.634*** |
|  | (0.0736) | (0.0811) | (0.0753) | (0.0701) | (0.0796) |
| Intervention group 1 (IG1) | 0.0136 | 0.144* | -0.0145 | -0.0171 | 0.0277 |
|  | (0.0859) | (0.0825) | (0.0911) | (0.116) | (0.0728) |
| Intervention group 2 (IG2) | -0.185 | -0.0378 | 0.00847 | -0.0826 | 0.213* |
|  | (0.132) | (0.173) | (0.158) | (0.213) | (0.128) |
| follow-up#IG1 | -0.136 | -0.177 | 0.0676 | -0.119 | -0.0280 |
|  | (0.112) | (0.115) | (0.100) | (0.116) | (0.101) |
| follow-up#IG2 | 0.171 | 0.137 | -0.0296 | 0.211 | -0.224 |
|  | (0.179) | (0.102) | (0.231) | (0.264) | (0.204) |
| female | -0.0735*** | 0.00295 | -0.00767 | 0.0270 | -0.0103 |
|  | (0.0233) | (0.0245) | (0.0208) | (0.0201) | (0.0222) |
| age | 0.00147* | -0.000251 | 0.00229** | -0.000119 | 0.000941 |
|  | (0.000767) | (0.000927) | (0.000934) | (0.000821) | (0.000804) |
| wealth quintile 2 | 0.0313 | 0.0693 | -0.0491 | 0.0272 | 0.0286 |
|  | (0.0884) | (0.0607) | (0.0666) | (0.0598) | (0.0639) |
| wealth quintile 3 | -0.0155 | 0.0869 | 0.0164 | -0.00271 | -0.0157 |
|  | (0.0924) | (0.0686) | (0.0859) | (0.0706) | (0.0691) |
| wealth quintile 4 | 0.0184 | 0.0120 | -0.0883 | -0.0962 | 0.0187 |
|  | (0.0899) | (0.0621) | (0.0800) | (0.0647) | (0.0706) |
| wealth quintile 5 | -0.108 | 0.0797 | -0.151* | -0.0804 | -0.0125 |
|  | (0.0918) | (0.0670) | (0.0852) | (0.0767) | (0.0746) |
| Western region | 0.474*** | 0.149** | 0.134** | -0.214* | 0.117** |
|  | (0.0661) | (0.0713) | (0.0557) | (0.113) | (0.0560) |
| less than primary | 0.108 | 0.0993 | -0.0307 | -0.128* | 0.114* |
|  | (0.0768) | (0.0985) | (0.0804) | (0.0656) | (0.0657) |
| primary | -0.122** | -0.0221 | 0.0216 | -0.0301 | 0.0308 |
|  | (0.0588) | (0.0630) | (0.0542) | (0.0525) | (0.0527) |
| junior secondary | -0.0696 | -0.0286 | -0.00963 | -0.0245 | 0.0131 |
|  | (0.0561) | (0.0477) | (0.0436) | (0.0416) | (0.0408) |
| senior secondary | -0.135** | -0.0610 | -0.00622 | -0.0265 | 0.00459 |
|  | (0.0660) | (0.0598) | (0.0669) | (0.0526) | (0.0567) |
| vocational/polytechnical | -0.150** | 0.00841 | -0.0520 | -0.0534 | 0.00689 |
|  | (0.0686) | (0.0774) | (0.0679) | (0.0688) | (0.0630) |
| higher | -0.0793 | -0.0497 | -0.118 | -0.131 | -0.329*** |
|  | (0.108) | (0.0959) | (0.113) | (0.0812) | (0.0922) |
| Christian | -0.0989 | -0.0480 | 0.107 | -0.0460 | -0.0391 |
|  | (0.0674) | (0.0752) | (0.0864) | (0.0626) | (0.0541) |
| pay for consultation | 0.0467 | -0.0263 | -0.0716* | 0.00375 | -0.191*** |
|  | (0.0445) | (0.0430) | (0.0412) | (0.0384) | (0.0412) |
| health status=same | 0.111** | -0.166*** | 0.0501 | 0.00405 | 0.0423 |
|  | (0.0447) | (0.0447) | (0.0423) | (0.0416) | (0.0466) |
| health status=worse | 0.0496 | -0.0547 | 0.0504 | -0.0496 | 0.137* |
|  | (0.0730) | (0.0736) | (0.0716) | (0.0708) | (0.0791) |
| pay for drugs | 0.152*** | -0.0470 | 0.136*** | 0.218*** | -0.00163 |
|  | (0.0392) | (0.0434) | (0.0340) | (0.0420) | (0.0341) |
| Constant | -0.269* | 0.0542 | -0.301** | 0.264** | 0.252*** |
|  | (0.146) | (0.116) | (0.141) | (0.131) | (0.0962) |
| Variance of intercept | 0.057*** | 0.068*** | 0.038*** | 0.192*** | 0.041*** |
|  | (0.015) | (0.014) | (0.008) | (0.037) | (0.009) |
| Observations | 6,013 | 6,013 | 6,013 | 6,013 | 6,013 |
| Number of groups | 64 | 64 | 64 | 64 | 64 |
| Log pseudolikelihood | -8340.4981 | -8340.4981 | -8444.9428 | -7746.1271 | -8098.8197 |

Note: Dependent variables are shown in column heading by model specification. Errors are clustered by facility. HC_trust 1 = Horizontal social capital – trust, HC_action = Horizontal social capital – action, VC_prov1 = Vertical social capital – provider 1, VC_prov2 = Vertical social capital – provider 2, VC_nhis = Vertical social capital – NHIS; * p<0.05; ** p<0.01; *** p<0.001

**Table D.3** The effect of the intervention on enrolling into health insurance (interaction effect with social capital factor are included)

|  | Model 1 | Model 2 | Model 3 | Model 4 | Model 5 |
| --- | --- | --- | --- | --- | --- |
|  | insured at follow-up | insured at follow-up | insured at follow-up | insured at follow-up | insured at follow-up |
|  |  |  |  |  |  |
| insured at baseline | 1.479*** | 1.485*** | 1.492*** | 1.488*** | 1.495*** |
|  | (0.180) | (0.182) | (0.179) | (0.181) | (0.180) |
| Intervention group 1 | 0.523*** | 0.569*** | 0.551*** | 0.594*** | 0.539*** |
|  | (0.183) | (0.188) | (0.186) | (0.195) | (0.191) |
| Intervention group 2 | 0.581** | 0.672** | 0.655*** | 0.679** | 0.836** |
|  | (0.292) | (0.278) | (0.254) | (0.274) | (0.358) |
| HC_trust | 0.0640 | -0.0886 | -0.0839 | -0.0842 | -0.0932 |
|  | (0.103) | (0.0841) | (0.0842) | (0.0833) | (0.0836) |
| IG1#c.HC_trust | -0.281 |  |  |  |  |
|  | (0.174) |  |  |  |  |
| IG2#HC_trust | -0.327 |  |  |  |  |
|  | (0.273) |  |  |  |  |
| HC_action | 0.108 | 0.177 | 0.114 | 0.118 | 0.117 |
|  | (0.0761) | (0.119) | (0.0753) | (0.0760) | (0.0758) |
| IG1#HC_action |  | -0.173 |  |  |  |
|  |  | (0.157) |  |  |  |
| IG2#HC_action |  | 0.0912 |  |  |  |
|  |  | (0.185) |  |  |  |
| VC_prov1 | 0.0324 | 0.0359 | 0.0184 | 0.0300 | 0.0305 |
|  | (0.0649) | (0.0656) | (0.102) | (0.0637) | (0.0656) |
| IG1#VC_prov1 |  |  | 0.0700 |  |  |
|  |  |  | (0.138) |  |  |
| IG2#VC_prov1 |  |  | -0.182 |  |  |
|  |  |  | (0.178) |  |  |
| VC_prov2 | 0.0560 | 0.0523 | 0.0527 | 0.181 | 0.0480 |
|  | (0.0940) | (0.0933) | (0.0939) | (0.133) | (0.0948) |
| IG1#VC_prov2 |  |  |  | -0.278 |  |
|  |  |  |  | (0.197) |  |
| IG2#VC_prov2 |  |  |  | -0.0412 |  |
|  |  |  |  | (0.222) |  |
| VC_nhis | 0.105 | 0.103 | 0.102 | 0.102 | 0.111 |
|  | (0.0850) | (0.0861) | (0.0862) | (0.0848) | (0.137) |
| IG1#VC_nhis |  |  |  |  | 0.0277 |
|  |  |  |  |  | (0.178) |
| IG2#VC_nhis |  |  |  |  | -0.312 |
|  |  |  |  |  | (0.308) |
| Western region | 0.431** | 0.431** | 0.430** | 0.417** | 0.425** |
|  | (0.169) | (0.168) | (0.169) | (0.175) | (0.171) |
| age | 0.0181*** | 0.0177*** | 0.0177*** | 0.0177*** | 0.0179*** |
|  | (0.00381) | (0.00380) | (0.00379) | (0.00386) | (0.00387) |
| female | 0.429*** | 0.424*** | 0.419*** | 0.423*** | 0.425*** |
|  | (0.103) | (0.104) | (0.105) | (0.104) | (0.104) |
| wealth quintile 2 | -0.0920 | -0.109 | -0.100 | -0.103 | -0.0973 |
|  | (0.175) | (0.176) | (0.176) | (0.176) | (0.173) |
| wealth quintile 3 | -0.126 | -0.134 | -0.129 | -0.111 | -0.113 |
|  | (0.219) | (0.217) | (0.219) | (0.214) | (0.216) |
| wealth quintile 4 | -0.0385 | -0.0474 | -0.0402 | -0.0363 | -0.0426 |
|  | (0.230) | (0.227) | (0.230) | (0.228) | (0.228) |
| wealth quintile 5 | -0.135 | -0.148 | -0.143 | -0.141 | -0.133 |
|  | (0.237) | (0.238) | (0.240) | (0.240) | (0.239) |
| pay for consultation | -1.359*** | -1.367*** | -1.378*** | -1.390*** | -1.380*** |
|  | (0.209) | (0.212) | (0.212) | (0.211) | (0.213) |
| pay for drugs | 0.978*** | 1.009*** | 0.997*** | 1.005*** | 1.009*** |
|  | (0.175) | (0.177) | (0.175) | (0.176) | (0.175) |
| health status=same | 0.112 | 0.104 | 0.0994 | 0.115 | 0.104 |
|  | (0.139) | (0.136) | (0.137) | (0.134) | (0.136) |
| health status=worse | 0.560* | 0.548* | 0.547* | 0.552* | 0.544* |
|  | (0.294) | (0.294) | (0.296) | (0.291) | (0.291) |
| less than primary | -0.232 | -0.258 | -0.240 | -0.231 | -0.255 |
|  | (0.264) | (0.265) | (0.267) | (0.267) | (0.267) |
| primary educ | -0.0883 | -0.0861 | -0.0911 | -0.0889 | -0.0969 |
|  | (0.237) | (0.236) | (0.236) | (0.236) | (0.236) |
| junior secondary | 0.165 | 0.158 | 0.169 | 0.167 | 0.168 |
|  | (0.171) | (0.170) | (0.171) | (0.169) | (0.170) |
| seniorsecondary | 0.356* | 0.354* | 0.351* | 0.353* | 0.355* |
|  | (0.192) | (0.191) | (0.191) | (0.187) | (0.193) |
| vocational/polytechnical | 0.749*** | 0.753*** | 0.757*** | 0.742*** | 0.759*** |
|  | (0.235) | (0.234) | (0.234) | (0.231) | (0.234) |
| higher | -0.167 | -0.123 | -0.129 | -0.139 | -0.124 |
|  | (0.392) | (0.394) | (0.390) | (0.394) | (0.392) |
| Christian | -0.121 | -0.121 | -0.117 | -0.141 | -0.144 |
|  | (0.257) | (0.254) | (0.264) | (0.253) | (0.257) |
| Constant | -3.018*** | -3.010*** | -3.008*** | -3.024*** | -3.009*** |
|  | (0.457) | (0.456) | (0.458) | (0.452) | (0.461) |
|  |  |  |  |  |  |
| Intercept variance | 0.319*** | 0.334*** | 0.334*** | 0.353*** | 0.346*** |
|  | (0.068) | (0.074) | (0.072) | (0.081) | (0.074) |
| Observations | 2,723 | 2,723 | 2,723 | 2,723 | 2,723 |
| Number of groups | 64 | 64 | 64 | 64 | 64 |
| Log pseudolikelihood | -1405.5631 | -1407.7705 | -1408.5248 | -1406.7335 | -1408.4805 |

Note: The dependent variable of each model specification is being insured at follow-up. This table is the basis for Figure D.1. Errors are clustered by facility. HC_trust 1 = Horizontal social capital – trust, HC_action = Horizontal social capital – action, VC_prov1 = Vertical social capital – provider 1, VC_prov2 = Vertical social capital – provider 2, VC_nhis = Vertical social capital – NHIS; * p<0.05; ** p<0.01; *** p<0.001

**Figure D.1**

Marginal effects of the two interventions at various social capital levels

Note: Marginal effects are computed from Table D.3.
